# A Red Fluorescent Protein-Based Probe for Detection of Intracellular Reactive Sulfane Sulfur

**DOI:** 10.3390/antiox9100985

**Published:** 2020-10-13

**Authors:** Zimai Li, Qingda Wang, Yongzhen Xia, Luying Xun, Huaiwei Liu

**Affiliations:** 1 State Key Laboratory of Microbial Technology, Shandong University, Qingdao 266237, China; lizimai@mail.sdu.edu.cn (Z.L.); wangqingda@mail.sdu.edu.cn (Q.W.); xiayongzhen2002@sdu.edu.cn (Y.X.); luying_xun@wsu.edu (L.X.); 2 School of Molecular Biosciences, Washington State University, Pullman, WA 99164-7520, USA

**Keywords:** sulfane sulfur, antioxidation, mCherry, mitochondria, *Saccharomyces cerevisiae*

## Abstract

Reactive sulfane sulfur, including persulfide and polysulfide, is a type of regular cellular component, playing an antioxidant role. Its function may be organelle-dependent; however, the shortage of probes for detecting organellar reactive sulfane sulfur has hindered further investigation. Herein, we reported a red fluorescent protein (mCherry)-based probe for specifically detecting intracellular reactive sulfane sulfur. By mutating two amino acid residues of mCherry (A150 and S151) to cysteine residues, we constructed a mCherry mutant, which reacted with reactive sulfane sulfur to form an intramolecular –S_n_– bond (*n* ≥ 3). The bond largely decreased the intensity of 610 nm emission (excitation at 587 nm) and slightly increased the intensity of 466 nm emission (excitation at 406 nm). The 466/610 nm emission ratio was used to indicate the relative abundance of reactive sulfane sulfur. We then expressed this mutant in the cytoplasm and mitochondria of *Saccharomyces cerevisiae*. The 466/610 nm emission ratio revealed that mitochondria had a higher level of reactive sulfane sulfur than cytoplasm. Thus, the mCherry mutant can be used as a specific probe for detecting reactive sulfane sulfur in vivo.

## 1. Introduction

Sulfane sulfur, including persulfide and polysulfide, is commonly present in both eukaryotic and prokaryotic cells [1]. Increasing studies have demonstrated that it has important physiological functions, including antioxidation, anti-inflammation, angiogenesis, and cell signaling [2,3,4,5,6]. However, the mechanisms underpinning its functions are still largely unknown. Cellular sulfane sulfur exists as hydrogen polysulfide (HS_n_H, *n* ≥ 2), organopolysulfide (RS_n_H, *n* ≥ 2), and dialkyl polysulfide (RS_n_R, *n* ≥ 3). They come in different reactivities with a rough order of HS_n_H > RS_n_H > RS_n_R [7,8]. HS_n_H and RS_n_H have both electrophilic and nucleophilic properties, and vividly react with reactive oxygen species (ROS), reactive nitrogen species (RNS), and signaling molecules at physiological pH (7.0~7.6). Therefore, they are usually referred to as reactive sulfane sulfur, which actually functions in biological systems. In contrast, RS_n_R is relatively inert at physiological pH, which may work as a reservoir of cellular sulfane sulfur [9,10,11,12].

The distribution of cellular sulfane sulfur shows heterogeneity at subcellular levels in eukaryotic cells, the peroxisomes and mitochondria are reported to have more reactive sulfane sulfur (HS_n_H and GS_n_H) than the cytoplasm of *Saccharomyces cerevisiae* [13]. Both organelles perform important roles in ROS homeostasis maintenance [14,15]. The heterogeneity suggests that reactive sulfane sulfur may function differently in each subcellular organelle. A recent research revealed that cysteine persulfide (Cys-SSH), which is produced by cysteinyl-tRNA synthetase 2 (CRS2) in mammalian mitochondria, is involved in mitochondria biogenesis and bioenergenesis [16]. Apart from this finding, little is known about the organellar sulfane sulfur. The shortage of specific methods for its dynamical detection has seriously hindered further investigation.

To address this challenge, we recently developed a green fluorescent protein (GFP)-based reactive sulfane sulfur detection probe, psGFP1.1, which reacts with hydrogen polysulfide and alters its fluorescence [13]. On the other hand, many widely used cell dyes and probes are based on the green range of fluorescence, and hence the GFP probe cannot be used simultaneously with them. In addition, peptone, yeast extract, and metal irons also have fluorescence in the green wavelength range. Some intracellular metabolites, such as flavin mononucleotide (FMN, λ_em,max_ = 525 nm and λ_ex,max_ = 450 nm), can disturb the detection as well [17]. Therefore, the application of psGFP1.1 is limited in certain circumstances. To expand the toolbox of the fluorescence protein-based sulfane sulfur detection probe, we herein developed the red fluorescent protein-based probe, psRFP. We expressed this probe in the cytoplasm and mitochondria of yeast cells, demonstrating that it is a useful tool for studying intracellular sulfane sulfur at a subcellular level. Together with psGFP1.1, it will contribute to the study of cellular sulfane sulfur.

## 2. Materials and Methods

### 2.1. Strains, Plasmids, and Compounds

*Escherichia coli* DH5α was used for plasmid construction. *E. coli* BL21(DE3) and *S. cerevisiae* BY4742 were used for protein expression. *E. coli* strains were grown in lysogeny broth (LB) medium. Kanamycin (50 μg/mL) was added when required. *S. cerevisiae* BY4742 was grown in SD-ura^-^ medium (synthetic defined minimal medium without uracil) [18]. Dithiothreitol (DTT), L-cystine, and L-cysteine hydrochloride monohydrate were purchased from BBI life sciences (Shanghai, China) Company. Trans-4,5-dihydroxy-1,2-dithiane (Oxidized DTT), sodium thiosulfate pentahydrate (Na_2_S_2_O_3_), sodium sulfite (Na_2_SO_3_), Isopropyl β-D-1-thiogalactopyranoside (IPTG), flavin mononucleotide (FMN), and reduced glutathione (GSH) were purchased from Sigma-Aldrich (Shanghai, China). HS_n_H and GSSH (glutathione persulfide) were prepared and quantified by following reported protocols [19,20].

### 2.2. Protein Mutation, Expression, and Purification

The gene encoding mCherry was ligated into the plasmid pET30a with a C-terminal His-tag. Amino acid mutations were introduced by a modified QuikChange™ method [21]. The obtained pET30a-mCherry and pET30a-psRFP were transformed into *E. coli* BL21(DE3). The recombinant strains were grown in LB at 37 °C with shaking (210 rpm) until the absorbance at 600 nm (OD_600_) reached about 0.6, and then 0.4 mM IPTG was added. The cells were further cultured at 25 °C with shaking (180 rpm) for 20 h. Cells were collected by centrifugation and broken open using a crusher SPCH-18 (Stansted Fluid Power Ltd.,London, UK). Protein purification was carried out with the nickel-nitrilotriacetic acid agarose resin (Invitrogen, New York, USA). SDS-PAGE analysis was performed to analyze the purity of finally obtained proteins. Buffer exchange of the purified proteins was performed with a PD-10 desalting column (GE Healthcare, New York, USA).

### 2.3. Characterization of psRFP

The purified protein (75 μM) was mixed with a reactant (200 μM H_2_O_2_, 200 μM DTT, 200 μM HS_n_H, 200 μM GSSH, 1 mM Na_2_SO_3_, or 1 mM Na_2_S_2_O_3_) in Tris-HCl buffer (50 mM, pH 7.4). The reaction was performed at room temperature for 1 h, and then the unreacted reactants were removed with the PD-10 desalting column. The fluorescence of the reacted protein was analyzed by using a RF-5301 PC spectrofluorophotometer (Shimadzu, Kyoto, Japan).

The absorbance of purified psRFP and mCherry was analyzed by using a UV-1800 spectrophotometer (Shimadzu, Kyoto, Japan). Equation (1) was used to calculate the quantum yield of psRFP:

*Φ_x_* = *(A_s_/A_x_)(F_x_/F_s_)(n_x_/n_s_)^2^* × *Φ_s_* (1)

In Equation (1), *Φ* is the quantum yield, *A* is the absorbance, *F* is the total fluorescent emission, *n* is the refractive index of the solvents used, *x* is the sample to be detected (psRFP), and *s* is the standard (mCherry). Equation (2) was used to calculate the extinction coefficient of psRFP:

*A* = *εbc* (2)

In Equation (2), *A* is the absorbance, *ε* is the extinction coefficient, *b* is the optical distance, and *c* is the protein concentration. The brightness of psRFP was calculated by *Φ* × *ε*.

Redox midpoint potential was detected by using redox-oxidation titrations. Reduced DTT (DTT_red_) and oxidized DTT (DTT_ox_) were used to prepare the redox potential buffers, in which the total concentration of DTT (DTT_red_ + DTT_ox_) was 100 mM and the pH was set to 7.0. By adjusting the DTT_red_ to DTT_ox_ ratio, we prepared five redox buffers with different redox potentials (−389, −360, −330, −300, and −271 mV). The completely HS_n_H-oxidized psRFP was prepared by reacting 4.4 μM purified psRFP with 300 μM HS_n_H at room temperature for 1 h. Unreacted HS_n_H was removed by a PD-10 desalting column. The completely HS_n_H-oxidized psRFP was added into the redox buffers and incubated at room temperature for 1 h. The 466/610 nm ratios were analyzed by RF-5301 PC and the redox midpoint potential of psRFP, $E_{m\left(psRFP\right)}^{0}$, was calculated using an Equation (3):

(3) $$E_{DTT}=E_{m\left(DTT\right)}^{0}-\frac{RT}{zF}\ln\frac{{\left[DTT\right]}_{red}}{{\left[DTT\right]}_{ox}}=E_{m\left(psRFP\right)}^{0}-\frac{RT}{zF}\ln\left(\frac{1-OxD_{psRFP}}{OxD_{psRFP}}\right)$$

In Equation (3), $E_{m\left(DTT\right)}^{0}$ is the standard redox midpoint potential of DTT at pH 7 (−330 mV), *z* is the number of transferred electrons, *F* is the Faraday constant (96,485 C mol^−1^), *R* is the gas constant (8.315 J K^−1^ mol^−1^), and *T* is the absolute temperature (298.15 K). *OxD_psRFP_* is the percentage of oxidized psRFP (psRFP_ox_) in the system, which was calculated using Equation (4):

(4) $$\;OxD_{psRFP}=\frac{\left[psRFP_{ox}\right]}{\left[psRFP_{ox}\right]+\left[psRFP_{red}\right]}=\frac{R-R_{red}}{R_{ox}-R_{red}}$$

In Equation (4), R_ox_ and R_red_ refer to the 466/610 nm ratios of completely HS_n_H-oxidized psRFP and DTT-reduced psRFP, respectively. R is the 466/610 nm ratio detected by RF-5301 PC. The obtained OxD_psRFP_ data were plotted against the redox potential data generated by DTT redox buffers, and the curve was fitted into Equation (3) to calculate the psRFP redox midpoint potential.

### 2.4. Liquid Chromatography Tandem Mass Spectrum (LC-MS/MS) Analysis of psRFP

The purified protein (75 μM) was reacted with 300 μM HS_n_H or DTT at 25 °C for 1 h. The reacted mixture was loaded onto a PD-10 desalting column to remove unreacted small molecules. The re-purified proteins (≤100 μg) were then mixed with 50 μL denaturing buffer (0.5 M Tris-HCl, 2.75 mM Ethylenediaminetetraacetic acid (EDTA), 6 M Guanadine-HCl, pH 8.1) and 50 μL 1 M iodoacetamide (IAM) in a dark place for 1 h. 360 μL of 25 mM NH_4_HCO_3_ was used to wash the mixture four times in a Microcon YM-10 K centrifugal filter unit (Millipore, Billerica, MA, USA). The washed proteins were then digested with trypsin by following a previously reported protocol [22].

The Prominence nano-LC system (Shimadzu, Kyoto, Japan) equipped with a custom-made silica column (75 μm × 15 cm) packed with 3 μm Reprosil-Pur 120 C18-AQ was used for the analysis. For the elution process, a 100 min gradient from 0% to 100% of solvent B (0.1% formic acid in 98% acetonitrile) at 300 nL/min was used, and solvent A was 0.1% formic acid in 2% acetonitrile. The eluent was ionized and electro-sprayed via LTQ-Orbitrap Velos Pro CID mass spectrometer (Thermo Scientific, Waltham, MA, USA), which run in data-dependent acquisition mode with Xcalibur 2.2.0 software (Thermo Scientific, Waltham, MA, USA). The spray voltage was of −3.0 and 3.0 kV in negative and positive modes, respectively. The ion transfer tube temperature was set at 300 °C. Full-scan mass spectra (from 400 to 1800 m/z) were detected in the Orbitrap with a resolution of 60,000 at 400 m/z.

### 2.5. Analysis of Intracellualr Sulfane Sulfur with psRFP

*E. coli* BL21(DE3) harboring pET30a-psRFP plasmid (Table 1) was grown in LB medium at 37 °C with shaking (210 rpm) for 5 h. 10 μM IPTG was added at the beginning of the cultivation. After the cultivation, *E. coli* cells were collected and washed twice with Tris-HCl buffer (50 mM, pH 7.4). *E. coli* cells were suspended in the Tris buffer at OD_600_ of 1 and incubated with 2 mM DTT or 200 μM sulfur-containing chemicals (HS_n_H, GSSH, cystine, or cysteine) at room temperature for 1 h. After the sulfur-containing chemical treatment, cells were washed twice with Tris-HCl buffer and cell concentration was adjusted to OD_600_ = 1, and then fluorescence of the cells were analyzed by a Synergy H1 microplate reader.

For *S. cerevisiae* in vivo experiments, YEPlac195 plasmid containing TEF1 promoter was used for psRFP expression. Two plasmids, YEPlac195-psRFP_cyt_ and YEPlac195-psRFP_mit_, were constructed. YEPlac195-psRFP_cyt_ was used to express psRFP in cytoplasm, in which the psRFP encoding gene was directly incorporated into YEPlac195, after the TEF1 promoter. YEPlac195-psRFP_mit_ was used to express psRFP in mitochondria, in which, the MLSARSAIKRPIVRGLATV leading peptide was fused to the *N*-terminus of psRFP. The two plasmids were transformed into *S. cerevisiae* BY4742. Transformants were selected from SD-ura^-^ medium. The localization of psRFP in mitochondria was tested with Mito Tracker^TM^ Green (Thermo Scientific) following the manufacturer’s introduction. Recombinant *S. cerevisiae* strains were cultured in SD-ura^−^ medium at 30 °C with shaking (210 rpm). The middle-log phased cells (OD_600_ = 1) were collected and washed twice with Tris-HCl buffer (50 mM, pH 7.4), and incubated with 2 mM DTT or sulfur-containing chemicals (200 μM HS_n_H, cystine, cysteine, Na_2_S_2_O_3_) at room temperature for 1 h. After the sulfur-containing chemical treatment, cells were washed twice with Tris-HCl buffer and cell concentration was adjusted to OD_600_ = 1. The fluorescence of cell suspensions was analyzed by using a Synergy H1 microplate reader.

## 3. Results

### 3.1. Design of the Reactive Sulfane Sulfur-Sensitive mCherry

**Figure 1:**
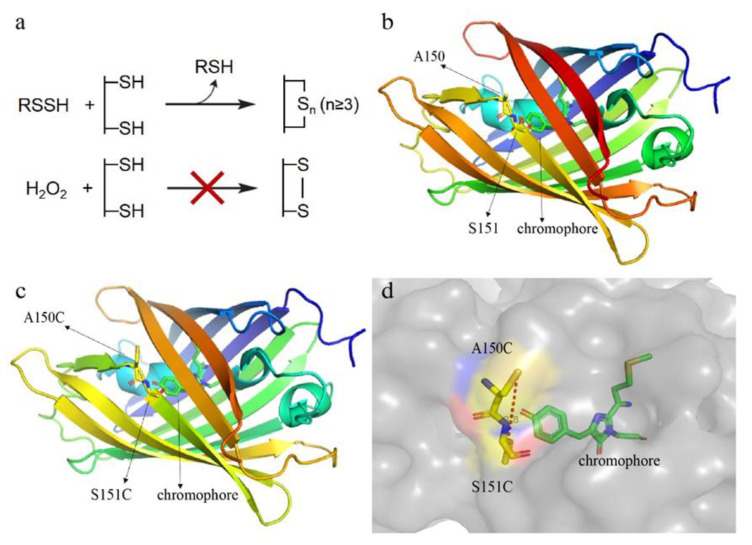
Designing principle and structure analysis of the reactive sulfane sulfur-sensitive mCherry. (**a**) Schematic representation of the reaction mechanisms. Reactive sulfane sulfur can transfer sulfane sulfur atom(s) to cysteine residues of the mCherry mutant to form an intramolecular –S_n_– (*n* ≥ 3)bond; however, the -S_2_- bond cannot be formed via H_2_O_2_ oxidation due to the long distance (>2.05 Å) between two thiol groups. (**b**) The locations of A150 and S151 in mCherry. The three-dimensional (3D) structure of mCherry was downloaded from the PDB website (PDB ID: 2H5Q). (**c**,**d**) The locations of C150 and C151, and the distance between their thiol groups in the mCherry mutant. The 3D structure of mCherry mutant was generated using SWISS-MODEL (http://swissmodel.expasy.org/) with mCherry as the template. The distance was analyzed with PyMol 1.5.0.3.

To avoid the disturbance caused by extracellular and intracellular compounds, we chose mCherry as the starting probe because its emission wavelength (λ_em,max_ = 610 nm) is out of the green range. The designing principle is to mutate two amino acid residues, whose locations are near the chromophore, to cysteine residues. Through controlling the distance of thiol groups of the two cysteine residues, a bridge with a –S_n_– (*n* ≥ 3) bond can be formed when the probe reacts with reactive sulfane sulfur (Figure 1a). The –S_n_– bond has π electrons, hence, it may affect the electron distribution of mCherry chromophore, thereby altering the excitation and/or emission wavelength of mCherry chromophore. Out of the consideration that –S_n_– (disulfide) bond may have the same effect, which is easily formed when the mutant probe encounters ROS, the distance between the two thiol groups should be longer than 2.05 Å, the length of a disulfide bond [23].

We used the three-dimensional (3D) structure of mCherry (PDB ID: 2H5Q) as a template and a homology modeling method to analyze the structure of mCherry mutant and found that converting A150 and S151 (Figure 1b) to cysteines might result in the potential probe. The thiol groups of this mutant were near to the chromophore (Figure 1c), and the calculated distance between them was 8.8 Å (Figure 1d).

### 3.2. Construction of the Hydrogen Polysulfide-Sensitive mCherry

**Figure 2:**
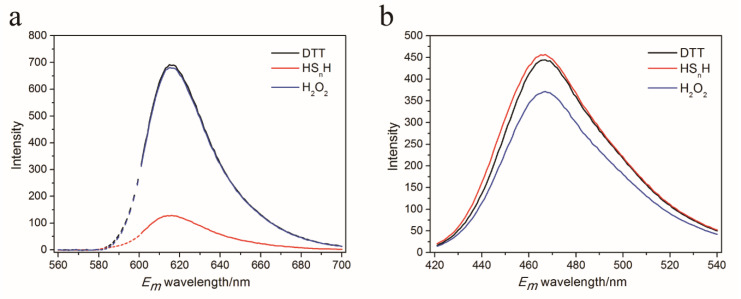
Emission spectra of Dithiothreitol (DTT), hydrogen polysulfide (HS_n_H), and H_2_O_2_-reacted psRFP at 587 nm excitation (**a**) and 406 nm excitation (**b**). 75 μM purified psRFP was mixed with a reactant (200 μM H_2_O_2_, 200 μM DTT, 200 μM HS_n_H) in Tris-HCl buffer (50 mM, pH 7.4). The reaction was performed at room temperature for 1 h, and then the unreacted reactants were removed with a PD-10 desalting column. The fluorescence of the reacted protein was analyzed by a RF-5301 PC spectrofluorophotometer.

We constructed the A150C-S151C double mutant and expressed it in *E. coli*. The mutant was fused with a C-terminus His-tag and was purified using the nickel column. SDS-PAGE analysis showed that the purity of the finally obtained protein was 74%. Fluorescence analysis indicated that the mutant had two obvious differences from the wild-type mCherry (wt): its 610 nm emission fluorescence (excitation at λ = 587 nm) dramatically decreased after it reacted with HS_n_H, while the fluorescence had no change after it reacted with H_2_O_2_ (Figure 2a). In comparison, the 610 nm emission fluorescence of wt was not affected by either HS_n_H or H_2_O_2_. It showed a new fluorescence spectra with λ_ex,max_ = 406 nm and λ_em,max_ = 466 nm. This fluorescence slightly increased when it reacted with HS_n_H but slightly decreased when it reacted with H_2_O_2_ (Figure 2b). This new fluorescence spectra was not observed from the wt. These results indicated that the A150C-S151C mutant has potentials to be used as a reactive sulfane sulfur detection probe. Hereafter, in this paper, we designate it as psRFP.

### 3.3. In Vitro Test of psRFP

**Figure 3:**
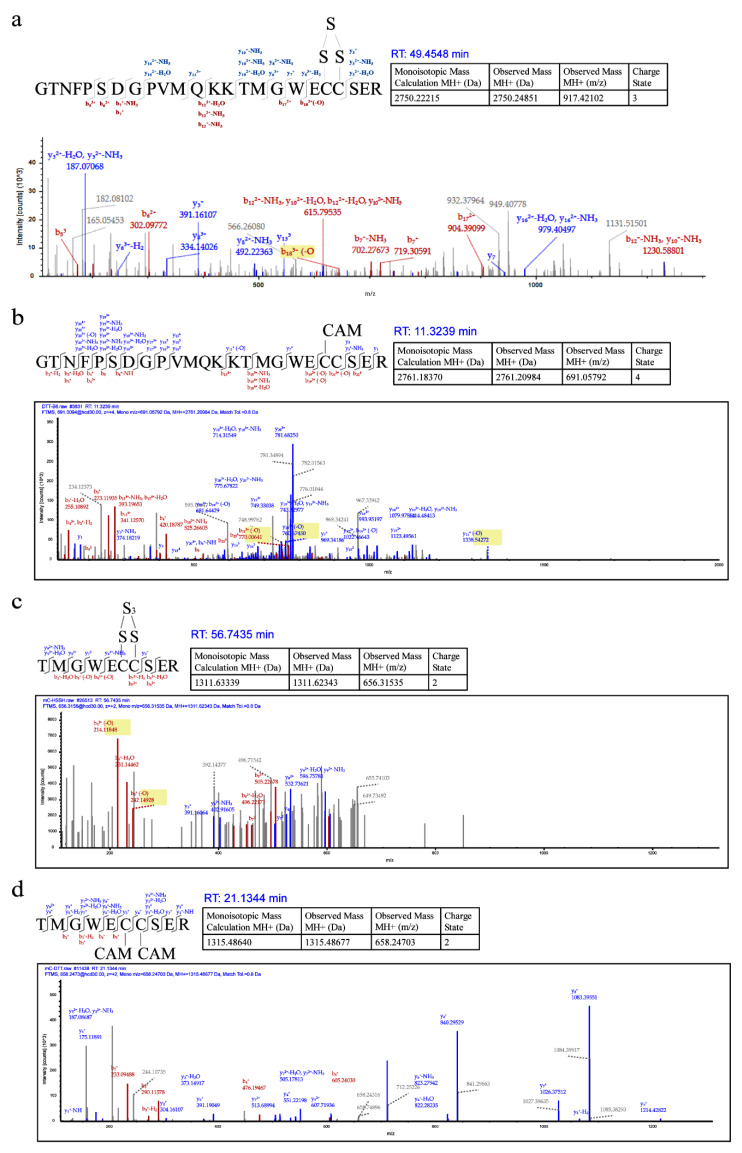
Liquid Chromatography tandem Mass Spectrum (LC-MS/MS) analysis of HS_n_H-reacted psRFP. (**a**) and (**c**) are from HS_n_H-reacted psRFP. (**b**) and (**d**) are from DTT-reacted psRFP.

We analyzed both HS_n_H-reacted psRFP and DTT-reacted psRFP with LTQ-Orbitrap Tandem MS. Two peptides containing 150C and 151C were detected in the HS_n_H-reacted psRFP, the first one had a 30.07 Da (+S, −2H) addition between the two cysteine residues, suggesting that a –S_3_– bond was formed (Figure 3a). Its counterpart was also found in the DTT-reacted psRFP, which has no sulfur addition between 150C and 151C (Figure 3b). The second peptide had a 94.2 (+3S, −4H) addition between 150C and 151C, suggesting that a –S_5_– bond was formed (Figure 3c). Its counterpart was also found in the DTT-reacted psRFP, which has no sulfur addition (Figure 3d).

**Figure 4:**
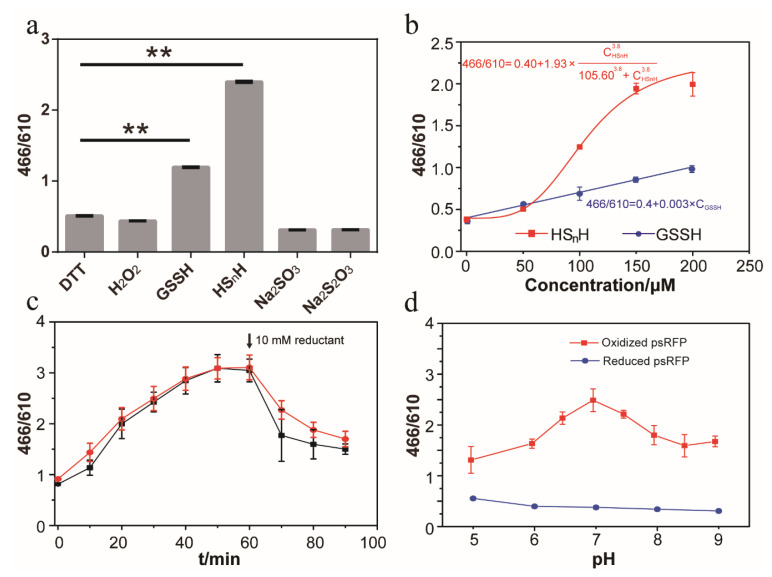
In vitro characterization of psRFP. (**a**) 466/610 nm emission ratios of reacted psRFP. Symbol ** indicates that the sample is significantly different (*p* < 0.01) from that of DTT-reacted psRFP. (**b**) Dose-dependent response of psRFP to glutathione persulfide (GSSH) and hydrogen polysulfide (HS_n_H). Hill equation was used to fit the data obtained from the psRFP-HS_n_H reaction and linear equation was used to fit the data obtained from the psRFP-GSSH reaction. (**c**) DTT (black line) and reduced glutathione (GSH) (red line) reduced the HS_n_H-reacted psRFP. (**d**) pH effects to the 466/610 nm emission ratios of reduced psRFP (reacted with DTT) and HS_n_H-oxidized psRFP (reacted with HS_n_H). For (**a**), 75 μM purified psRFP was mixed with a reactant (200 μM H_2_O_2_, 200 μM DTT, 200 μM HS_n_H, 200 μM GSSH, 1 mM Na_2_SO_3_, or 1 mM Na_2_S_2_O_3_) in Tris-HCl buffer (50 mM, pH 7.4). The reaction was performed at room temperature for 1 h, and then the unreacted reactants were removed with a PD-10 desalting column. For (**b**), the reaction conditions were the same, except for that the concentrations of GSSH and HS_n_H were different. For (**c**), 75 μM purified psRFP was mixed with 200 μM HS_n_H in Tris-HCl buffer (50 mM, pH 7.4). The reaction was performed at room temperature. An increase of the 466/610 nm emission ratio was observed. When 10 mM DTT (red line) or GSH (black line) was added at 60 min, the ratio quickly decreased in 10 min. For (**d**), the reaction conditions were the same as in (**a**), except for that the reaction buffer was changed to phosphate buffer (67 mM, pH 5.0, 6.0, or 6.5) or Tris-HCl buffer (50 mM, pH 7.0, 7.5, 8.0, 8.5, or 9.0). The fluorescence of the reacted proteins were all analyzed by a RF-5301 PC spectrofluorophotometer. The data of (**a**–**d**) were from three independent repeats and shown as mean value ± standard deviation (SD).

Since the HS_n_H reaction resulted in both the increase of 466 nm emission and the decrease of 610 nm emission, we used the 466/610 nm emission ratio to indicate the proportion of HS_n_H-oxidized psRFP (containing a –S_n_– bond) to reduced psRFP (containing –SH groups) in the reacted psRFP samples. For the DTT-reacted psRFP sample, the ratio was 0.51. For the H_2_O_2_-reacted sample, the ratio was 0.44 (Figure 4a), indicating that ROS-related reactions had minimal disturbance to the 466/610 nm emission ratio of psRFP. For the HS_n_H-reacted psRFP sample, the ratio was 2.39. We also used other sulfur-containing chemicals that commonly exist in cells to react with psRFP. Glutathione persulfide (GSSH) caused a mild change to the 466/610 nm emission ratio, while sulfite (Na_2_SO_3_) and thiosulfate (Na_2_S_2_O_3_) showed no effect. These results indicated that psRFP preferred to react with reactive sulfane sulfur.

The above reactions were conducted in a condition that the concentrations of reactants were much higher than that of psRFP. Next, we used gradient concentrations of HS_n_H and GSSH to react the psRFP. The values of the 466/610 nm emission ratio were linearly dependent on the concentrations of the reactants (Figure 4b). We used the Hill equation to fit the data obtained from the psRFP-HS_n_H reaction. Parameters showed that the K_m_ is 105.6 and the Hill coefficient is 3.8. Under the applied condition, the detection range for HS_n_H is 50~200 μM. We used the linear equation to fit the data obtained from the psRFP-GSSH reaction. Parameters showed that the slope is 0.003 and the intercept is 0.4. psRFP showed higher sensitivity toward HS_n_H than GSSH, probably because HS_n_H is more active than GSSH [7].

When psRFP was mixed with HS_n_H, the 466/610 nm emission ratio gradually increased and reached the maximum at 50 min, and when 10 mM DTT was added at 60 min, the ratio quickly decreased in 10 min (Figure 4c). This observation indicated that the HS_n_H-oxidized psRFP was reducible by DTT. Since GSH is an important antioxidant in many cells, we checked whether it can reduce the HS_n_H-oxidized psRFP or not. Results showed that when 10 mM GSH was mixed with the HS_n_H-oxidized psRFP, the 466/610 nm emission ratio was also decreased, although with less degree compared to that caused by 10 mM DTT (Figure 4c).

We tested the pH effect to the 466/610 ratio of reduced and HS_n_H-oxidized psRFP. The 466/610 ratio of reduced psRFP (DTT-treated) was a little higher at pH 5.0 than that at pH 9.0, but in the range of pH 6~8, the ratio change was negligible (Figure 4d). For the HS_n_H-oxidized psRFP, the 466/610 ratio was significantly higher at neutral pH (7.0) than at acidic or alkaline pH. In the tested pH range, the 466/610 ratios of HS_n_H-oxidized psRFP were always higher than those of reduced psRFP, indicating that psRFP can be used to detect sulfane sulfur in different pH conditions.

Using the redox-oxidation titration method and DTT (whose redox midpoint potential is −330 mV) solutions as the calibrating redox buffer, we measured the redox midpoint potential of psRFP to be −363 mV at pH 7.0 and 25 °C. We also measured the quantum yield (Φ), extinction coefficient (ε), and brightness of psRFP (λ_em_ = 610 nm, in reduced form, Table 2). These photophysical parameters were all lower than those of mCherry (Table 2).

### 3.4. In Vivo Test of psRFP

**Figure 5:**
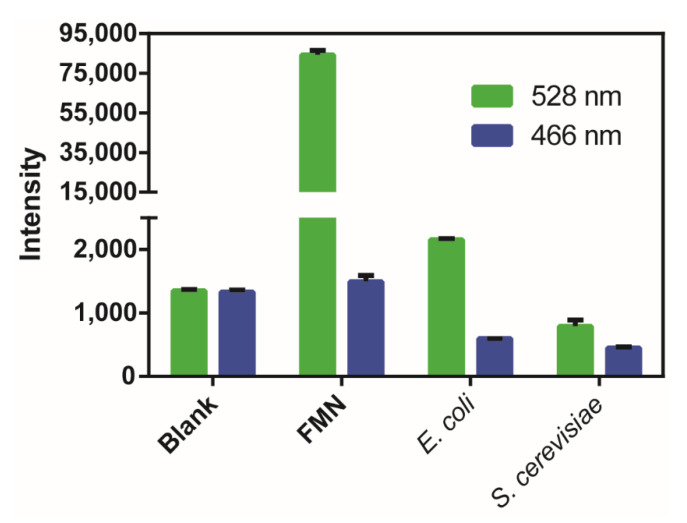
Fluorescence analysis of flavin mononucleotide (FMN), *E. coli*, and *S. cerevisiae* cells. A Synergy H1 microplate reader was used and the 528 nm emission fluorescence was excited at 485 nm, and the 466 nm emission fluorescence was excited at 406 nm. Blank is the Tris-HCl buffer (50 mM, pH 7.4). FMN (50 μM), *E. coli*, and *S. cerevisiae* cells (OD_600_ = 1) were suspended in the Tris-HCl buffer (50 mM, pH 7.4). The data were from three independent repeats and shown as mean value ± SD.

To evaluate the cellular interference to the blue fluorescence at 466 nm, we analyzed the flavin mononucleotide (FMN), the common intracellular chromophore that has strong 528 nm emission fluorescence (excited at 485 nm). Its 466 nm emission fluorescence (excited at 406 nm) was about 60-fold lower that of 528 nm and was only slightly higher than the Tris-HCl buffer (Figure 5). These results indicated that FMN cannot interfere with the mCherry-based probe. We also analyzed the fluorescence of *E. coli* and *S. cerevisiae* cells and observed that again, the 466 nm fluorescence is relatively weak; hence, the cellular interference to the psRFP probe is limited.

**Figure 6:**
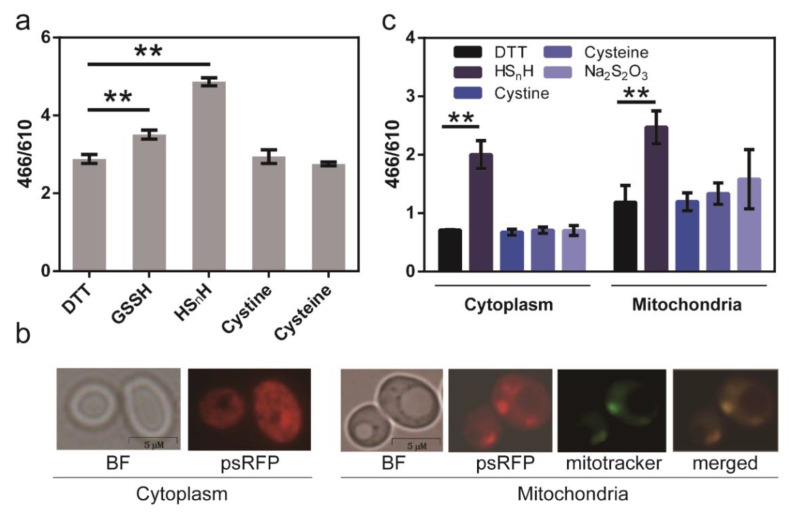
Using the psRFP to analyze intracellular reactive sulfane sulfur. (**a**) psRFP was expressed in cytoplasm of *E. coli* BL21(DE3). *E. coli* cells (OD_600_ = 1) were collected and washed twice with Tris-HCl (50 mM, pH 7.4), and then incubated with 2 mM DTT or 200 μM sulfur-containing chemicals (HS_n_H, GSSH, cystine, or cysteine) at room temperature for 1 h. The data were from three independent repeats and shown as mean value ± SD. Symbol ** indicates that the sample is significantly different (*p* < 0.01) from DTT-treated cells. (**b**) psRFP was expressed in *S. cerevisiae* BY4742 cytoplasm or mitochondria. Photos were captured with an Olympus fluorescence microscope (IX83, 63×). (**c**) *S. cerevisiae* cells (OD_600_ = 1) were collected and washed twice with Tris-HCl (50 mM, pH 7.4), and then incubated with 2 mM DTT or sulfur-containing chemcials (200 μM HS_n_H, cystine, cysteine, Na_2_S_2_O_3_) at room temperature for 1 h. The fluorescence of both *E. coli* and *S. cerevisiae* cells was analyzed with a Synergy H1 microplate reader. The data were from three independent repeats and shown as mean value ± SD. Symbol ** indicates the sample is significantly different (*p* < 0.01) from the DTT-treated sample.

Next, we used psRFP to detect the intracellular sulfane sulfur of *E. coli* BL21 (DE3). *E. coli* cells expressing psRFP were incubated with different sulfur-containing chemicals, and the 466/610 ratios were measured. Both HS_n_H- and GSSH-treated cells had obviously higher 466/610 ratios than DTT-treated (Figure 6a), demonstrating that sulfane sulfur-treated cells had more intracellular reactive sulfane sulfur, as expected. In comparison, cystine or cysteine treatment did not lead to obvious increases of the 466/610 ratio, indicating that they had no or minimum effect to the level of intracellular reactive sulfane sulfur.

We then used psRFP to analyze the intracellular reactive sulfane sulfur of *S. cerevisiae* BY4742. It was expressed in BY4742 cytoplasm and mitochondria. For expression in cytoplasm, psRFP contained no signal peptide. For expression in mitochondria, psRFP was fused with an N-terminal signal peptide MLSARSAIKRPIVRGLATV. The recombinant yeast cells were treated with different sulfur-containing chemicals, and the 466/610 ratios were measured (Figure 6b). HS_n_H treatment led to an obvious increase of 466/610 ratios in both cytoplasm and mitochondria. Cysteine and thiosulfate caused slight increases of 466/610 ratios in mitochondria, but not in cytoplasm. In addition, the 466/610 ratios in mitochondria were generally higher than those in cytoplasm, suggesting that mitochondria should be the major production organelle in *S. cerevisiae*.

## 4. Discussion

Although some chemically synthesized probes have been developed for detection of reactive sulfane sulfur in vivo, only several of them are commercially available, making their application limited [26,27,28,29,30,31]. In this study, we developed an easily available, mCherry-based, and reaction-reversible probe that enables real-time analysis of intracellular reactive sulfane sulfur. The working mechanism of psRFP is when, reacting with reactive sulfane sulfur, an intramolecular –S_n_– bond can be formed between its two cysteine residues. The 610 nm emission fluorescence intensity decreased from 690 to 110 after it reacted with HS_n_H (detected by the RF-5301 PC spectrofluorophotometer), the amplitude of the change was 580. This amplitude is large enough to be detected in a common fluorophotometer. Therefore, it is suitable for reactive sulfane sulfur detection, as confirmed by in vivo experiments. We also observed that psRFP had a new fluorescence peak (λ_ex,max_ = 406 nm and λ_em,max_ = 466 nm), which is slightly affected by the –S_n_– bond. The 466/610 nm emission ratio can directly indicate the proportion of HS_n_H-oxidized psRFP to reduced psRFP; therefore, this probe can be used in both in vitro and in vivo detection of cellular reactive sulfane sulfur. It is noteworthy that the oxidized psRFP can be reduced by GSH; therefore, the reactive sulfane sulfur level detected by psRFP may to some extent reflect the GSH level in the cell. Actually, GSH can directly react with reactive sulfane sulfur and their complex reactions have been intensely studied [1,2,8,9,10]. The level of reactive sulfane sulfur itself is a compromised consequence of the complex reactions among sulfane sulfur, GSH, and hydrogen sulfide.

Compared with the GFP-based probe psGFP1.1, psRFP has similar detection range and sensitivity. The reaction time of psRFP with HS_n_H is longer (~50 min) than that of psGFP1.1 with HS_n_H (~15 min). The most important difference between them is that psRFP has no emission fluorescence at the ~500 nm range; hence, it can be used simultaneously with other cell dyes or probes having green fluorescence. In addition, its fluorescence signal is less disturbed by cell components. A disadvantage of psRFP is that its brightness (λ_em_ = 610 nm) is much lower than that of mCherry (Table 2). In contrast, psGFP1.1 shows no significant brightness decrease compared to its parental GFP [9]. The low brightness is not a severe drawback when psRFP is applied in analysis of *E. coli* or *S. cerevisiae*, because high concentrations of psRFP can be achieved via using plasmids for its expression. However, when we tried to use it for analysis of the human colorectal carcinoma cell HCT116, we observed that the fluorescence signals were too low to give a meaningful indication (data not shown). The reason why 150C-S151C double mutations cause this brightness decrease is still unknown. To develop a mammalian cell-friendly probe, the same mutation strategy might be applied to other red fluorescence proteins, and the mutant whose brightness is less changed should be preferred.

## 5. Conclusions

A mCherry mutant was constructed by mutating two amino acid residues (A150 and S151) to cysteine residues. Thiol groups of these two cysteine residues can react with reactive sulfane sulfur to form an intramolecular –S_n_– bond (*n* ≥ 3). The bond largely decreases the intensity of 610 nm emission (excitation at 587 nm) and slightly increases the intensity of 466 nm emission (excitation at 406 nm). Therefore, the 466/610 nm emission ratio can be used to indicate the relative abundance of reactive sulfane sulfur. By expressing the mCherry mutant in the cell, in vivo reactive sulfane sulfur can be dynamically detected.

## Figures and Tables

**Table 1:** Strains and plasmids used in this study.

Strains and Plasmids	Relevant Characteristics/Purposes	References
**Strains**		
*S. cerevisiae* BY4742	*MATα his3Δ1 leu2Δ0 lys2Δ0 ura3Δ0*	A gift from Prof. Jin Hou
*E. coli DH5a*	*supE44, AlacU169 (q80lacZAM15), hsdR17, recA1, endA1, gyrA96,thi-1, relA1*. For cloning and plasmid construction	Lab stock
*E. coli BL21(DE3)*	*F-ompT hsdSB (rB-mB-) gal ( λ1857 ind1 Sam7 nin5 lacUV5 T7gene1) dcm*. For expression and protein purification	Lab stock
**Plasmids**		
YEplac195-psGFPcyt	YEplac195 containing psRFP. Under control of TEF1 promoter	This study
YEplac195-psGFPmit	YEplac195 containing an N-ternimus sigal peptide ^a^-psRFP. Under control of TEF1 promoter	This study
pET30a-psRFP	pET30a containing psRFP. Under control of T7 promoter for expression and protein purification	This study
pET30a-mCherry	pET30a containing mCherry. Under control of T7 promoter for expression and protein purification	This study

^a^ The peptide sequence is MLSARSAIKRPIVRGLATV.

**Table 2:** Photophysical parameters of mCherry and psRFP.

	Φ	ε (M^−1^·cm^−1^ × 10^−3^)	Brightness	$E_{m}^{0}$ (mv)
MCherry ^1^	0.22	72	15.84	n.d. ^2^
psRFP	0.0076	5.4	0.04	−363

^1^ The parameters were referred to in References [24,25]. ^2^ mCherry has no cysteine residues; hence, its redox potential was not analyzed.
